# *Addendum:* Bastos, J.C.S.; Kohn, L.K.; Fantinatti-Garboggini, F.; Padilla, M.A.; Flores, E.F.; da Silva, B.P.; de Menezes, C.B.A.; Arns, C.W. Antiviral Activity of *Bacillus* sp. Isolated from the Marine Sponge *Petromica citrina* against Bovine Viral Diarrhea Virus, a Surrogate Model of the Hepatitis C Virus. *Viruses* 2013, *5*, 1219-1230

**DOI:** 10.3390/v5071682

**Published:** 2013-07-11

**Authors:** Juliana Cristina Santiago Bastos, Luciana Konecny Kohn, Fabiana Fantinatti-Garboggini, Marina Aiello Padilla, Eduardo Furtado Flores, Bárbara Pereira da Silva, Cláudia Beatriz Afonso de Menezes, Clarice Weis Arns

**Affiliations:** 1 Laboratório de Virologia, Departamento de Genética Evolução e Bioagentes, Instituto de Biologia/Universidade Estadual de Campinas-UNICAMP, Cx. Postal 6109, CEP 13083-970 , Campinas/SP/Brazil; E-Mails: lucianakohn@gmail.com (L.K.K.); fabianaf@cpqba.unicamp.br (F.F.-G.); ma_padilla2001@yahoo.com.br (M.A.P.); barbarapds84@gmail.com (B.P.d.S); clbeatriz2003@gmail.com (C.B.A.d.M.); clarns@gmail.com (C.W.A.); 2 Centro de Ciências Rurais, Departamento de Medicina Veterinária Preventiva. Universidade Federal de Santa Maria (UFSM) CEP 97105-900 - Santa Maria/RS/Brazil; E-Mail: eduardofurtadoflores@gmail.com

The authors wish to add the following Acknowledgments and completed Table 1 to their paper published in *Viruses* [1], doi:10.3390/v5051219, website: http://www.mdpi.com/1999-4915/5/5/1219.

## Acknowledgments

This research was supported by grant 2009/53320-7, São Paulo Research Foundation (FAPESP) and LKK would like to thank CNPq for a post-doctoral scholarship (503259/2011-1).

**Table 1:** General characteristics of the extracts evaluated for antiviral activity.

Extract	Sponge	Genbank Access number	Identification	Inhibition percentage (%)
**B511**	*Chelonaplysilla erecta*	HQ433229	*Exiguobacterium* sp.	33
**B515**	*Chelonaplysilla erecta*	HQ433231	*Vibrio* sp.	25
**B555**	*Petromica citrina*	KF294467	*Bacillus* sp.	98
**B565**	*Petromica citrina*	KF294469	*Bacillus* sp.	37
**B584**	*Petromica citrina*	KF294468	*Bacillus* sp.	98
**B616**	*Petromica citrina*	KF294466	*Bacillus* sp.	90

Characteristics of the extracts evaluated for antiviral activity. Name of the each extract evaluated, sponge from which the microorganism was obtained, Genbank access number, identification of the microorganism from which the extract was obtained, and inhibition percentage obtained on antiviral assay.
